# Supplementary material for: Optimizing in vitro fertilization in four Caribbean coral species
Source: PeerJ. 2025 Jan 31;13:e18918. doi: 10.7717/peerj.18918 (PMC11789651; doi:10.7717/peerj.18918)
Supplement: Supplemental Information 1 [file peerj-13-18918-s001.docx]

**Supplemental Materials**

**Optimizing *In Vitro* Fertilization in Four Caribbean Coral Species**

Valérie F Chamberland^1,2,3*‡^, Matthew-James Bennett^1‡^, Tania Doblado Speck^1^, Kelly RW Latijnhouwers^1,2,3^, Margaret W Miller^1^,

^1^ SECORE International, Miami, FL, USA

^2^ CARMABI Foundation, Willemstad, Curaçao

^3^ Department of Freshwater and Marine Ecology, Institute for Biodiversity and Ecosystem Dynamics, University of Amsterdam, Amsterdam, The Netherlands

^‡^Equal first authors

^*^Corresponding Author: [v.chamberland@secore.org](about:blank)

**Table S1.** Date and time of spawning and gamete collections as well as bundle break up and oocyte diameter for all coral species included in this study. Times and days relative to the full moon (d AFM) are given for Curaçao (GMT -4). Oocyte diameter data were collected during previous coral spawning events (Chamberland VF, unpub. data).

**Table S2.**  Test conditions and results for all three assays testing fertilization success in function of **a** sperm concentration (Log_10_ cell mL^-1^), **b** gamete age (hr after spawning (AS)), and **c** gamete co-incubation period (hr). Fertilization success (%) is the average proportion of fertilized eggs across replicates (n); SD = standard deviation; SE = standard error; [Sperm] = sperm concentration (cell mL^-1^) in the assays.
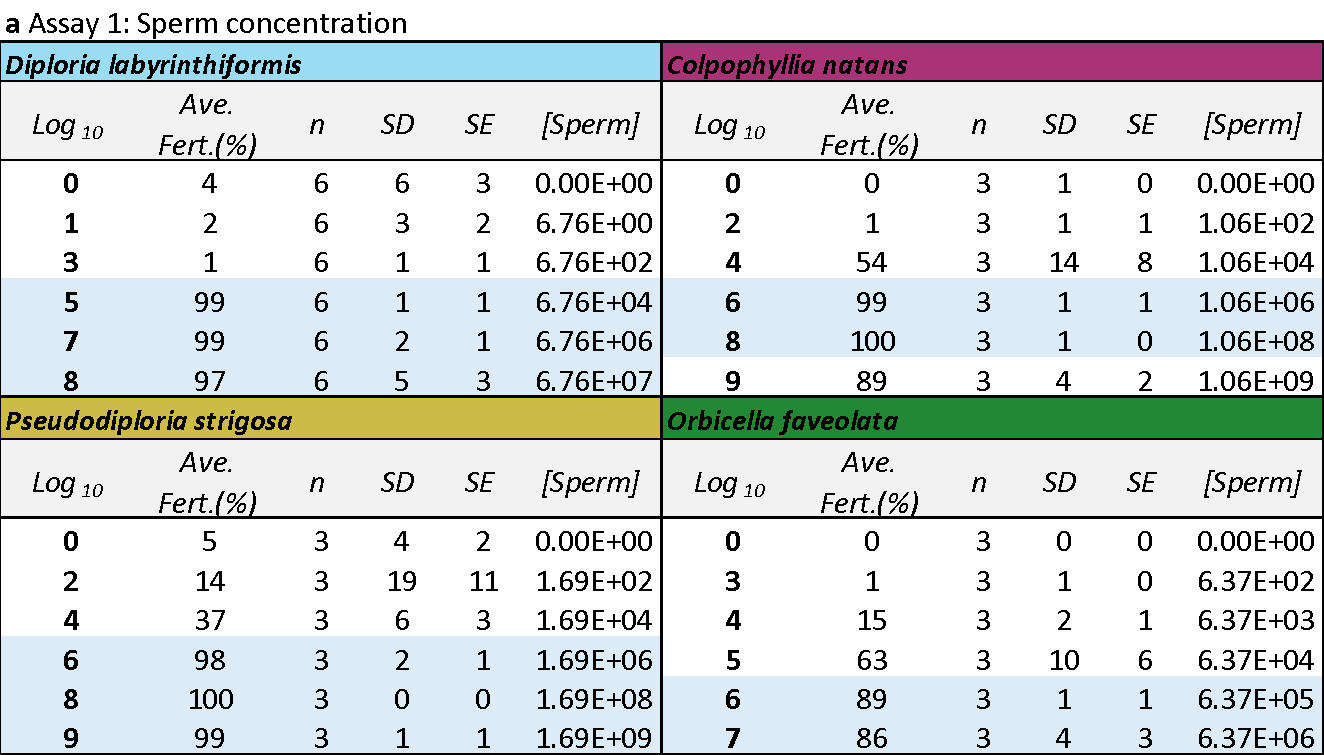


**Table S2.** Continued.


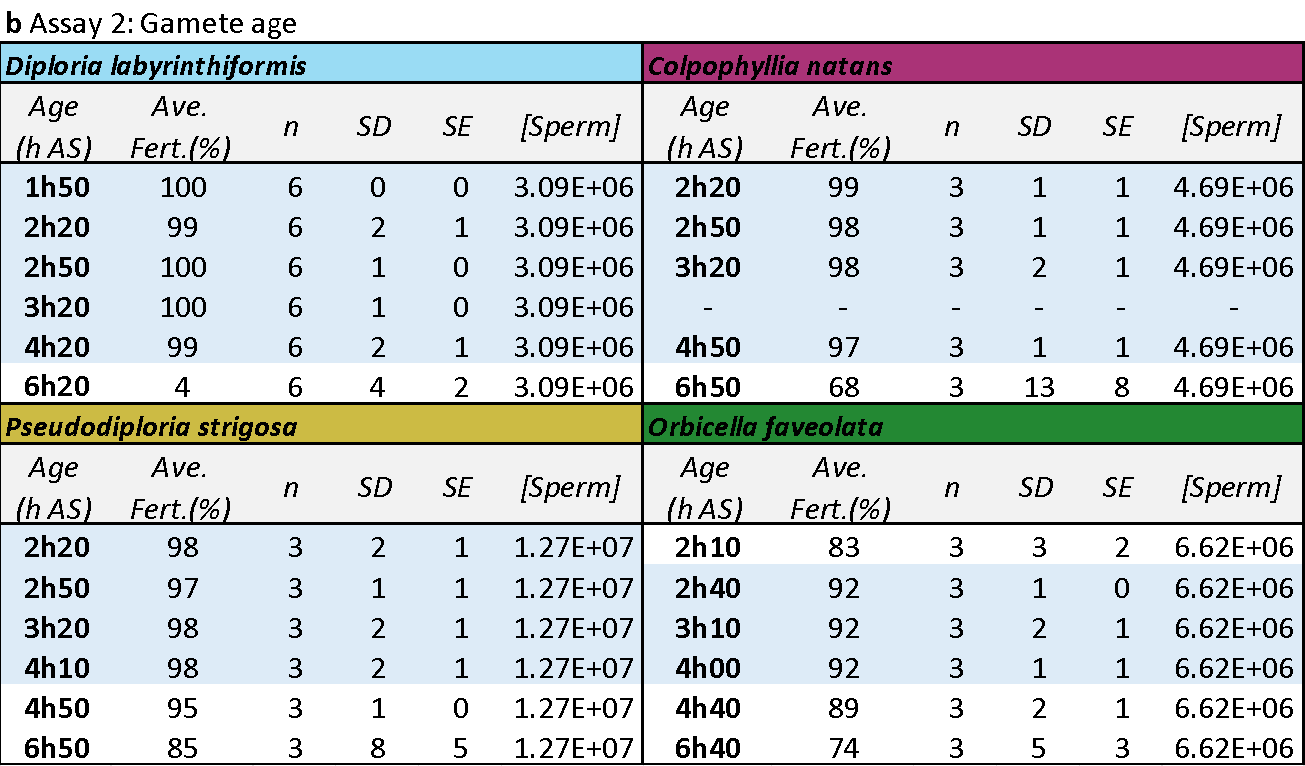


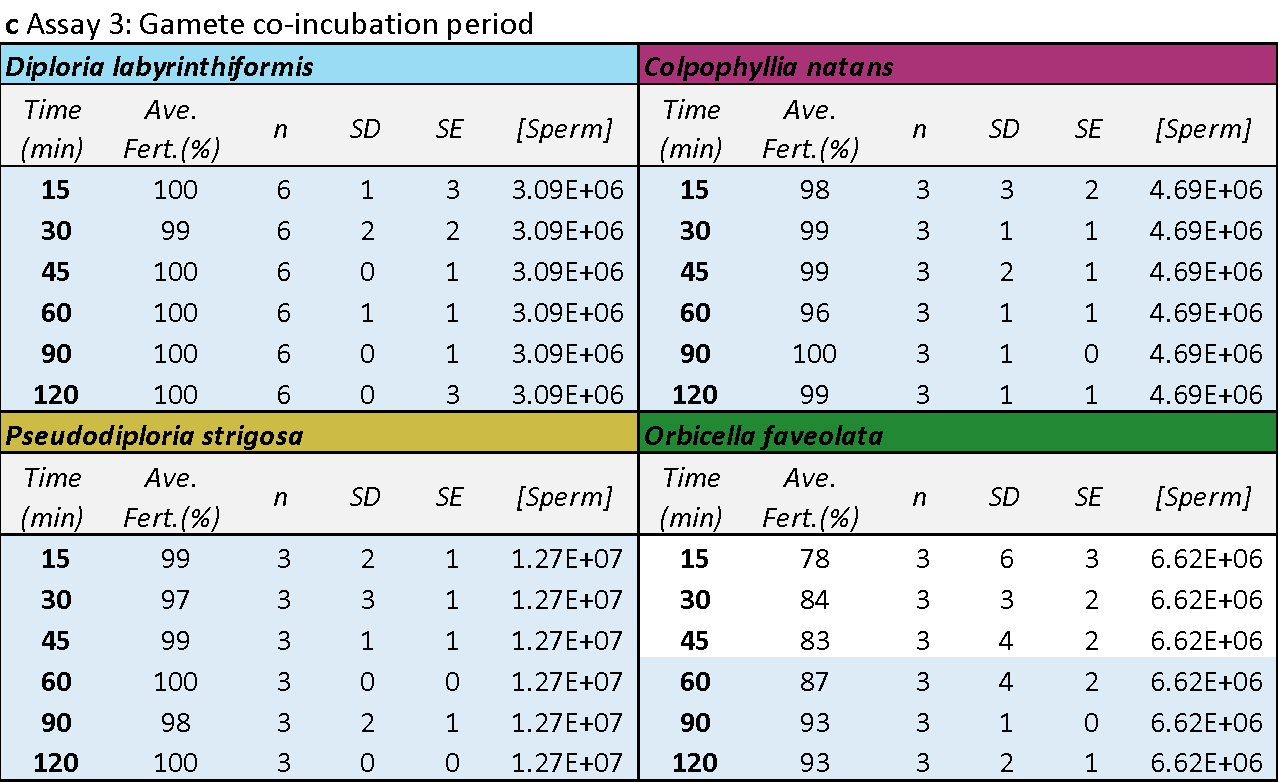


**Table S3.** Statistical results for all three fertilization assays. Data was first analyzed using Kruskal-Wallis non-parametric tests to determine statistical differences in fertilization success among treatments for each assay. When significant differences were detected, Dunn’s *post hoc* pairwise comparisons were performed to identify different treatment groups. Significant results are in bold.

**Table S3.**  Continued.

**Table S3.**  Continued.


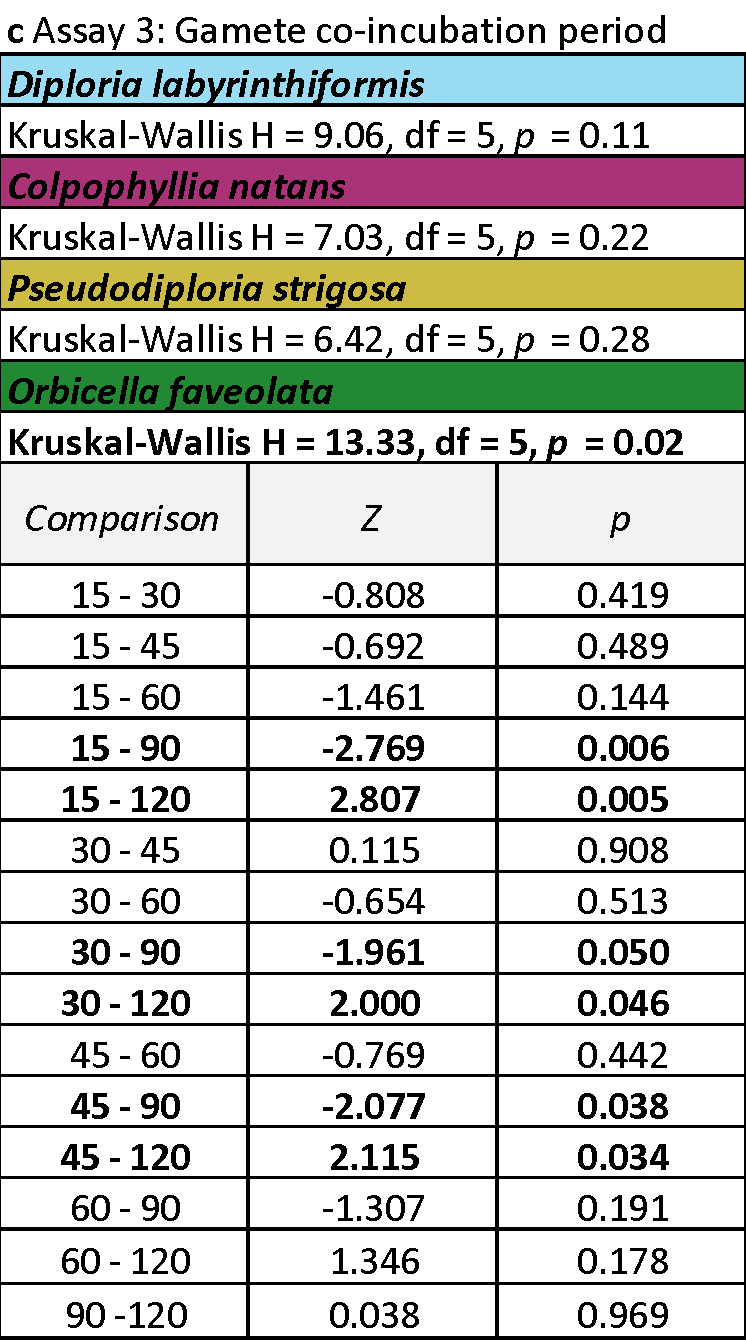


**Table S4.** Summary of available information on the influence of sperm concentration on fertilization success in scleractinian corals. Experimental conditions (*i.e.*, co-incubation period and gamete age) are provided where available.

**Table S4.** Continued.
